# Pregnancy Outcome after Varenicline Exposure in the First Trimester

**DOI:** 10.1155/2014/263981

**Published:** 2014-02-03

**Authors:** Yusuf Cem Kaplan, Nihal Olgac Dündar, Burcu Kasap, Barıs Karadas

**Affiliations:** ^1^Department of Pharmacology, Teratology Information Service, Faculty of Medicine, Izmir Katip Celebi University, 35280 Izmir, Turkey; ^2^Department of Pediatric Neurology, Faculty of Medicine, Izmir Katip Celebi University, 35280 Izmir, Turkey; ^3^Department of Obstetrics and Gynaecology, Faculty of Medicine, Mugla Sitki Kocman University, 48000 Mugla, Turkey

## Abstract

To the best of our knowledge this is the first case report describing exposure to varenicline, an *α*_4_ *β*_2_ nicotinic acetylcholine partial receptor agonist used for smoking cessation therapy in pregnancy. A 29-year-old multiparous woman with an unplanned pregnancy has used varenicline 2 mg/day unintentionally yet regularly 4 weeks from her last menstrual period. Fetal ultrasound performed at each trimester, detailed anomaly scan, and fetal echocardiography which were performed at the 22nd gestational week showed normal fetal growth with no malformations. The patient delivered a healthy baby at the 38th week of gestation with normal Apgar score and physical examination findings. Age-appropriate physical and neurological development of the child has been observed for 6 months. Although it is not possible to draw definitive conclusions, this case report may contribute to the current available limited data regarding the safety of varenicline use in pregnancy.

## 1. Introduction

Cigarette smoking during pregnancy is associated with a range of adverse pregnancy outcomes such as spontaneous abortion, placental abruption and previa, preterm birth, small for gestational age, and fetal and neonatal death [1, 2]. Although it is ideal to stop smoking before the pregnancy, quitting smoking even during pregnancy, particularly by the 16th week, is reported to have beneficial effects on birth weight and cognitive functions in children [1].

Nicotine replacement therapy (NRT), bupropion, and partial nicotine receptor agonists such as varenicline are pharmacological treatment options for smoking cessation in the general population [3]. Human pregnancy outcome data regarding the use of NRT and bupropion are available to some extent; however data regarding the use of varenicline during pregnancy is only limited to preclinical animal studies.

To our knowledge this case report is the first human pregnancy outcome data regarding varenicline exposure in the first trimester of pregnancy.

## 2. Case Report

A 29-year-old multiparous caucasian woman with an unplanned pregnancy has been exposed to varenicline 1 mg twice a day unintentionally 4 weeks from her last menstrual period (LMP). Limited rodent data regarding varenicline did not permit a definitive risk assessment; however, after the risk communication with the patient, she decided to continue pregnancy with perinatology follow-up. She received folic acid supplementation between weeks 4 and 12. Fetal ultrasound (USG) performed at each trimester showed normal fetal growth with no malformations. A detailed anomaly scan and fetal echocardiography were performed at the 22nd gestational week and revealed normal development of fetus. The patient delivered a healthy baby girl via cesarean section at the 38th week of gestation. First- and fifth-minute Apgar scores were 9 and 10, respectively. The infant was the second child of nonconsanguineous parents. The baby's weight was 3130 g, length was 49 cm, and head circumference was 34 cm with normal percentiles and the baby did not show any physical examination abnormalities postnatally. Neurological examination had normal findings including posture and muscle tone, newborn reflexes, and deep tendon reflexes.

In the follow-up controls, at the ages of 2, 4, and 6 months we saw an age-appropriate developed child with a normal internal and neurological status. Social contact, fine motor, language, and gross motor skill categories measured with Denver II Developmental Screening Test adapted and standardized for Turkish children at the fifth and sixth month were compatible with her age.

## 3. Discussion

Varenicline was developed in 1997 as a selective *α*_4_ *β*_2_ nicotinic acetylcholine receptor (nAChR) partial agonist. The mechanism of effect is to provide a continuous and moderate release of mesolimbic dopamine and counteract the withdrawal symptoms caused by low dopamine release during the smoking cessation period. Additionally, varenicline blocks the effects of a subsequent nicotine challenge on dopamine release from the mesolimbic neurons which has a central role in the development of nicotine dependence [4]. It gained approval by The Food and Drug Administration (FDA) as a prescription-only aid for smoking cessation in 2006 [5].

A recent meta-analysis showed that varenicline increases the chances of successful smoking cessation when compared with placebo [5]. Varenicline was demonstrated to increase abstinence rates at 6 months or longer compared with bupropion in nonpregnant populations [3]. Limited data is available regarding varenicline versus NRT; however it was suggested to be more effective than NRT because it had been found to be more efficacious than bupropion [5].

Experiences regarding the use of varenicline in pregnancy are only limited to preclinical animal studies. Placental transfer of varenicline was shown in both rats and rabbits. Varenicline administration to pregnant rats failed to increase congenital anomalies at a maternal dose of 15 mg/kg/day which is about 36 times the human dose. No adverse effects of varenicline on fertility were identified in adult rats; however offspring showed an increase in auditory startle reflex and female offspring showed a decrease in fertility. A higher dose, 30 mg/kg/day, which was administered to pregnant rabbits also did not induce congenital anomalies; however a decrease in body weight of fetuses was detected [6]. It was suggested that potential neurotoxicity of varenicline is needed to be tested against nicotine exposure and placebo in animal studies [1]. An observational phase 4 clinical trial is underway to assess the safety of varenicline during pregnancy [3].

Current evidence considers behavioral therapy and patient education as the first-line therapies for smoking cessation in pregnant patients. For pharmacological intervention due to the failure of therapy, NRT seems to have more pregnancy safety and effectiveness data than bupropion and varenicline. A meta-analysis reported that neither of the latter two could be recommended for use in pregnancy because of the lack of adequate studies [2]. However, it was also suggested in a study that use of NRT and bupropion might be considered after a risk-benefit assessment and discussion with the patient [3].

To our knowledge, our case is the first human report regarding varenicline exposure in pregnancy. It is always hard to build causality and reach conclusions from case reports, particularly in assessing drug exposures in pregnancy. However, this case may be considered as a small contribution to the available limited safety data until epidemiological studies are completed. Undoubtedly, newborns would have the greatest health benefit if smoking is quitted prior to pregnancy.
